# Catalyst-Less and Transfer-Less Synthesis of Graphene on Si(100) Using Direct Microwave Plasma Enhanced Chemical Vapor Deposition and Protective Enclosures

**DOI:** 10.3390/ma13245630

**Published:** 2020-12-10

**Authors:** Rimantas Gudaitis, Algirdas Lazauskas, Šarūnas Jankauskas, Šarūnas Meškinis

**Affiliations:** Institute of Materials Science, Kaunas University of Technology, K. Baršausko St. 59, LT-51423 Kaunas, Lithuania; rimantas.gudaitis@ktu.lt (R.G.); algirdas.lazauskas@ktu.edu (A.L.); sarunas.jankauskas@ktu.lt (Š.J.)

**Keywords:** graphene, direct plasma synthesis, microwave plasma enhanced chemical vapor deposition

## Abstract

In this study, graphene was synthesized on the Si(100) substrates via the use of direct microwave plasma-enhanced chemical vapor deposition (PECVD). Protective enclosures were applied to prevent excessive plasma etching of the growing graphene. The properties of synthesized graphene were investigated using Raman scattering spectroscopy and atomic force microscopy. Synthesis time, methane and hydrogen gas flow ratio, temperature, and plasma power effects were considered. The synthesized graphene exhibited n-type self-doping due to the charge transfer from Si(100). The presence of compressive stress was revealed in the synthesized graphene. It was presumed that induction of thermal stress took place during the synthesis process due to the large lattice mismatch between the growing graphene and the substrate. Importantly, it was demonstrated that continuous horizontal graphene layers can be directly grown on the Si(100) substrates if appropriate configuration of the protective enclosure is used in the microwave PECVD process.

## 1. Introduction

Graphene is a monolayer or several layers of hexagonally shaped carbon atoms [1]. This 2D carbon nanomaterial has achieved considerable interest due to the huge mobility of electrons and holes, optical transparency, flexibility, and chemical inertness [1,2,3]. Graphene is already considered to be a new transparent conductor [4,5], a monolayer alternative to the Schottky contact metals [6,7], and even an active layer of semiconductor devices [8,9,10,11,12]. Graphene-based transistors [8], diodes [6,7], photodetectors [9,10,11], and solar cells [12,13,14] are also meaningful in this context.

A complicated graphene transfer process is one of the main limitations preventing the broader use of graphene in semiconductor device technology. In this instance, graphene is grown on the copper of nickel catalytic foils [15]; followed by the complicated graphene transfer onto the required dielectric or semiconductor substrates. During the transfer process, different adsorbates can contaminate graphene [16]. Additionally, the transfer process can cause wrinkled or rippled surface morphology of graphene [17]. In this case, the control of the graphene film or graphene-semiconductor interface properties becomes a tricky task. Graphene can be synthesized on a silicon carbide (SiC) substrate if appropriate vacuum heating conditions are used [18]. No catalytic metals are necessary in this case [18]. However, the present use of SiC as a semiconductor is mainly limited by some segments of high-power electronics [19]. SiC apart, it was shown recently that direct graphene synthesis on the semiconductor or dielectric surfaces is possible via the use of plasma-enhanced chemical vapor deposition (PECVD) [20]. In this case, plasma activation of the chemical vapor deposition is mandatory. It ensures enhanced dissociation of the plasma species during the graphene synthesis process. However, plasma-related ion and electron bombardment of the growing graphene surface is detrimental. It results in the creation of defects and may even make the etching process prevail over the graphene growth [21]. Therefore, remote plasma is used for direct graphene synthesis.

Nevertheless, remote plasma mode is unavailable in the most conventional microwave and inductively coupled plasma-based PECVD units. However, there are few studies on catalyst-less and transfer-less horizontal graphene synthesis using direct PECVD. In this process, the growing graphene film is additionally protected by some plasma shielding. Notably, the [21] sample was enclosed in a metal cage with a honeycomb mesh shield, while in [22], a copper-foam-based Faraday cage was applied. Direct graphene synthesis on insulating substrates such as glass [22], sapphire [21], quartz [21] plates, as well as thermally deposited SiO_2_, Al_2_O_3_, MnO_2_, HfO_2_, and TiO_2_ films [21] was demonstrated. However, for many devices, graphene synthesis on semiconductor substrates is necessary. Monocrystalline Si(100) is still the most often used substrate for the fabrication of microelectronic devices, solar cells, and different photodiodes. Therefore, catalyst-less and transfer-less graphene synthesis on Si(100) using a direct microwave plasma system was considered in the present study. 

In this paper, the samples were protected from direct plasma action using several different configurations of protective enclosures. The enclosures’ design was varied, taking into account two processes: eliminating the unwanted direct plasma effects and flow of the reactive carbon, hydrocarbon, and hydrogen species towards the substrate. The protective enclosure should screen the substrate from direct plasma. At the same time, gas flows are changed due to the presence of the enclosure. Herein, we wanted to know to what extent we can further suppress excessive direct plasma action by reducing the enclosure’s top hole size or removing the top holes above a substrate, and to what extent we can reduce protective enclosure design complexity. Synthesis parameters and their influence on the graphene structure were analyzed thoroughly. We have shown that graphene can be synthesized on the Si(100) substrate in a one-step process using a combination of the direct plasma and differently shaped enclosures. It was revealed that even a very simple enclosure design consisting of a single rectangular steel sheet without holes could be used as protective shielding. 

## 2. Materials and Methods

The direct transfer-less synthesis of graphene was performed by the microwave PECVD system Cyrannus (Innovative Plasma Systems (Iplas) GmbH, Troisdorf, Germany). A methane and hydrogen gas mixture was used as a source of carbon and hydrogen. The hydrogen plasma was ignited until the heater reached the target temperature. Hydrogen gas flow and plasma power were the same as in the graphene growth process (Table 1). Methane gas was introduced when the temperature necessary for graphene synthesis was reached. The growth process was conducted in one-step without a separate nucleation stage. 

**Figure 1:**
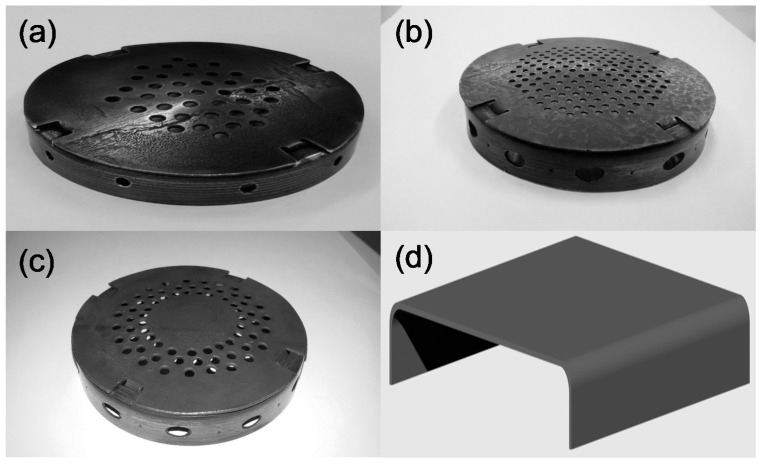
Protective enclosures used for direct synthesis of the graphene on Si(100): the first (1st) enclosure (top hole size 3.5 mm) (**a**), the second (2nd) enclosure (top hole size 2 mm) (**b**), the third (3rd) enclosure (top hole size 3.5 mm, no holes at the center) (**c**), the fourth (4th) enclosure (enclosure height 5 mm) (**d**).

Monocrystalline Si(100) (UniversityWafer Inc., South Boston, MA, USA) was applied as a substrate for the direct synthesis of graphene. No additional wet chemical cleaning of the substrate was performed. Special enclosures protected the sample from excessive plasma action. Four steel enclosures of different designs were used (Figure 1) to protect the sample from excessive plasma action. Three circular enclosures (1st–3rd) had holes of different diameters and pattern arrangements on the top. The 4th enclosure had a much simpler design and consisted of a rectangular steel sheet folded in two places. 

Technological parameters such as plasma power, CH_4_/H_2_ gas flow ratio, pressure, temperature, and time were varied. The graphene direct synthesis conditions can be found in Table 1. 

Raman scattering spectra of the synthesized samples were acquired using the Raman spectrometer inVia (Renishaw, Wotton-under-Edge, UK). The excitation wavelength was 532 nm. The excitation laser beam power was 1.5 mW. The ratio of 2D and G peak intensities (I_2D_/I_G_ ratio) was estimated to evaluate the number of graphene layers [23]. The I_D_/I_G_ peak intensity ratio was calculated to estimate the defect density of graphene [24,25]. Additionally, the positions of G and 2D peaks (Pos(G) and Pos(2D)) were also taken into account. Table S1 shows the possible relations between the Raman scattering spectra parameters mentioned above and the number of graphene layers, stress, doping, and defect density. The spectra were measured in several different places on the sample. The average values and standard deviation of the different Raman scattering spectra parameters were calculated for each sample. 

The surface morphology of the selected graphene layers was investigated using atomic force microscopy (AFM) at several different places on a sample. The measurements were done at room temperature in ambient air. The NanoWizardIII atomic force microscope (JPK Instruments, Bruker Nano GmbH, Berlin, Germany) was used. A v-shaped silicon cantilever operating in a contact mode was applied, as this mode is less sensitive to the possible presence of adsorbed species. The cantilever’s spring constant was 3 N/m, the tip curvature radius was 10.0 nm, and the cone angle was 20°. A 2 µm × 2 µm AFM scan area was chosen to reveal small graphene layer features. The SurfaceXplorer and JPKSPM Data Processing software (version spm-4.3.13, JPK Instruments) were applied for data analysis. 

## 3. Results

### 3.1. Raman Spectra of Directly Synthesized Graphene

In the present study, graphene was synthesized on the Si(100) substrates using microwave PECVD. Firstly, it is important to note that the direct synthesis of graphene on the Si(100) was not possible when the protective enclosure was not used in the plasma discharge zone during the microwave PECVD process. In this case, only the direct plasma interacting with the substrate was obtainable, which suppressed the graphene’s growth. 

**Figure 2:**
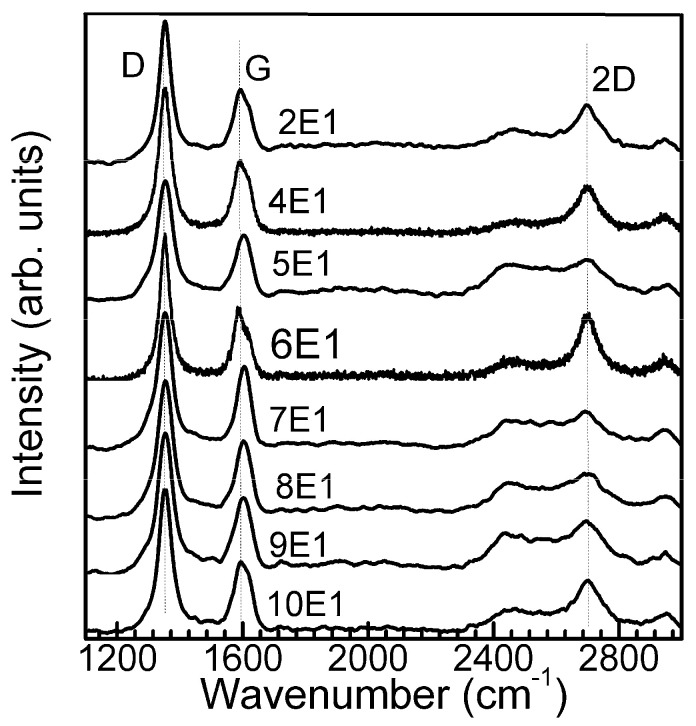
Typical Raman scattering spectra of graphene directly synthesized on Si(100).

In another instance, the sample was protected by the enclosure to prevent excessive direct plasma interaction with the substrate. Protective enclosures with several different designs were used as plasma shielding (Figure 1). In this case, the direct synthesis of graphene on the Si(100) substrate was successful. The recorded Raman scattering spectra of the samples were typical for graphene (Figure 2) [23,24,25,26,27,28,29,30,31,32,33,34,35,36,37,38,39,40,41,42,43]. Characteristic G and 2D peaks as well as defects related peaks (D peak as well as less intensive D+D″ and D+D′ bands [39,40]) were observed. No separate D′ peak at ~1620 cm^−1^ was observed in all cases. It is noteworthy that, in the Raman spectra of directly synthesized graphene, the D peak is always observed [20,21,22]. That is the main difference from the graphene synthesized by chemical vapor deposition (CVD) on catalytic foil and afterwards transferred onto the target substrate. This difference is mainly related to the presence of many grain boundary defects related to the nanocrystalline nature of directly synthesized graphene.

### 3.2. Effect of Synthesis Conditions and Enclosure Design on the Graphene Structure

The influence of the several key technological synthesis parameters (i.e., protective enclosure design, plasma power, methane and hydrogen gas flow ratio, pressure, temperature, and synthesis time) on the growth and structure of synthesized graphene was investigated. The 1st enclosure had 3.5 mm size holes on the top, while for the 2nd enclosure, the hole size was decreased to 2 mm. The 3rd enclosure had 3.5 mm size holes on the top, but no holes at the center (Figure 1).

**Figure 3:**
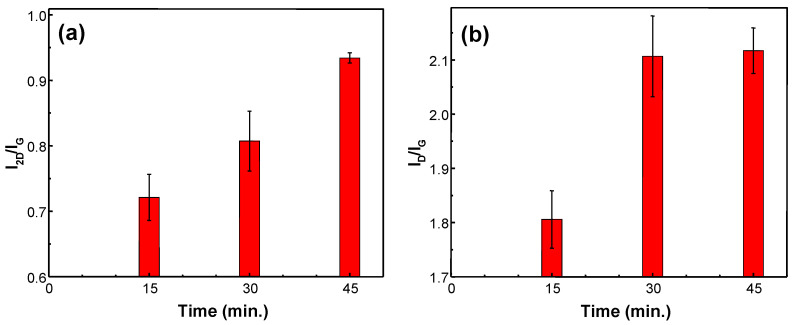
I_2D_/I_G_ (**a**) and I_D_/I_G_ (**b**) ratios of graphene synthesized at different process time (15 min, 30 min, 45 min). In all cases, every other process parameter is kept constant (H_2_ gas flow 150 sccm sccm, CH_4_ gas flow 50 sccm, power 1.2 kW, pressure 30 mBar, temperature 900 °C). The 1st enclosure was used.

Firstly, the effect of the synthesis time was considered (Figure 3). The graphene was already formed after 15 min of the microwave PECVD process. It was determined that after this time mark graphene gets thinner, and more defects are promptly introduced into the graphene structure: the increase in I_2D_/I_G_ from ~0.72 to ~0.93 (Figure 3a) and the increase in I_D_/I_G_ from ~1.8 to ~2.1 (Figure 3b). The other experiments’ synthesis time was chosen by taking into account these results (30 min).

**Figure 4:**
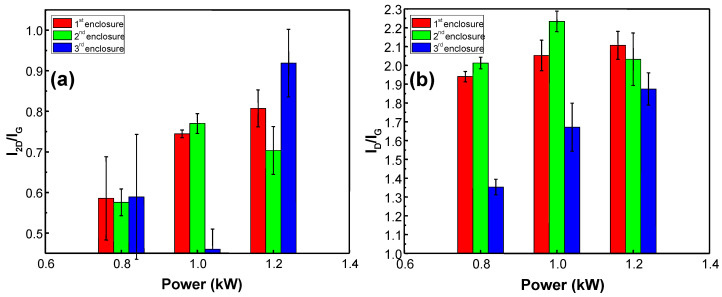
I_2D_/I_G_ (**a**) and I_D_/I_G_ (**b**) ratios of graphene synthesized using different plasma power (0.8 kW, 1.0 kW, 1.2 kW) and enclosures. In all cases, every other process parameter is kept constant (H_2_ gas flow 150 sccm sccm, CH_4_ gas flow 50 sccm, pressure 30 mBar, temperature 900 °C, time 30 min).

Figure 4 shows the I_2D_/I_G_ and I_D_/I_G_ ratios of graphene synthesized using different plasma power and enclosures. As it can be seen in Figure 4a, plasma power effect varies for different enclosures. The I_2D_/I_G_ ratio of graphene synthesized using the 1st enclosure increases with plasma power. This result implies that the number of graphene layers is reduced with an increase in plasma power. No clear dependence was observed for the 2nd and the 3rd enclosure. However, in all cases, the I_2D_/I_G_ ratio of the graphene synthesized using 1.2 kW power was higher than the I_2D_/I_G_ ratio of the graphene synthesized using 0.8 kW power. The I_D_/I_G_ ratio increased with plasma power for the 1st and the 3rd enclosure (Figure 4b). No clear dependence of the I_D_/I_G_ ratio on plasma power was observed for graphene synthesized using the 2nd enclosure.

**Figure 5:**
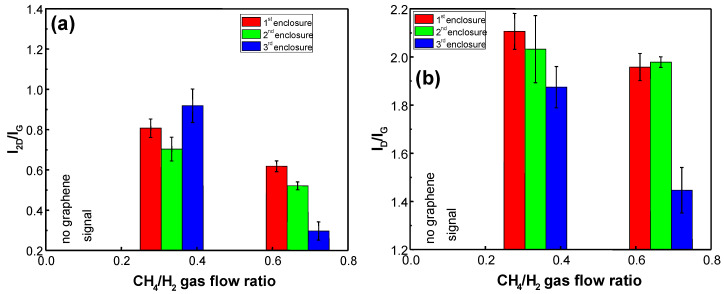
I_2D_/I_G_ (**a**) and I_D_/I_G_ (**b**) ratios of graphene synthesized using different flow ratio (0.11, 0.33, 0.67) CH_4_/H_2_ mixture of gas and enclosures. In all cases, every other process parameter is kept constant (power 1.2 kW, pressure 30 mBar, temperature 900 °C, time 30 min).

Figure 5 shows the I_2D_/I_G_ and I_D_/I_G_ ratios of graphene synthesized using different CH_4_/H_2_ gas flow ratio mixtures and enclosures. A too low CH_4_/H_2_ gas flow ratio (i.e., 0.11) was not sufficient to initiate the growth of graphene on the Si(100) substrate. The further increase in the methane flow and decrease in the hydrogen flow resulted in the increase in the number of graphene layers, as evident from the I_2D_/I_G_ ratio decrease (Figure 5a). It was also found that the I_D_/I_G_ ratio decreased with the increase in the CH_4_/H_2_ gas flow ratio for the 1st and the 2nd enclosure (Figure 5b). 

**Figure 6:**
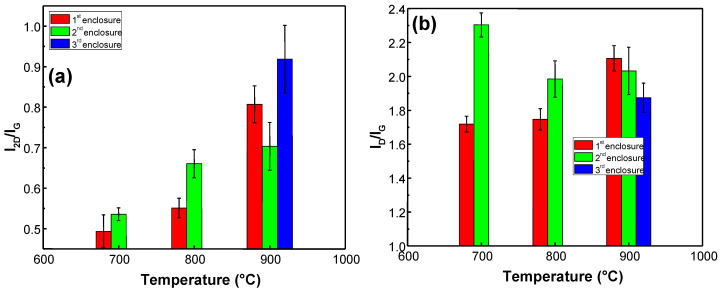
I_2D_/I_G_ (**a**) and I_D_/I_G_ (**b**) ratios of graphene synthesized using different temperatures (700 °C, 800 °C, 900 °C) and several enclosures. In all cases, every other process parameter is kept constant (H_2_ gas flow 150 sccm sccm, CH_4_ gas flow 50 sccm, power 1.2 kW, pressure 30 mBar, time 30 min).

Figure 6 shows the I_2D_/I_G_ and I_D_/I_G_ ratios of graphene synthesized using different temperatures and enclosures. It is important to note that no graphene growth was observed at 600 °C. As it can be seen in Figure 6a, the I_2D_/I_G_ ratio increases with the process temperature. Thus, the number of graphene layers grown decreases with the increase in temperature when the microwave PECVD process is performed in the range of 700–900 °C. For graphene synthesized using the 1st enclosure, the I_D_/I_G_ ratio increases with temperature (Figure 6b). However, the highest density of defects was observed for the graphene synthesized using the 2nd enclosure at 700 °C. 

Altogether, it was revealed that the most crucial graphene direct synthesis parameter in our case is the CH_4_/H_2_ gas flow ratio. A too low ratio results in no graphene synthesis. The number of graphene layers increases with methane flow when the ratio is substantially large for graphene synthesis. At the same time, defect density decreases. This is valid for all studied enclosures. When the temperature is too low, graphene does not grow. Subsequently, the temperature increase results in the decrease in graphene layer thickness. However, no typical behavior regarding defect density can be found. The plasma power effects are the least clear. The possible physical mechanisms hidden behind these results will be considered in Section 4.2 and Section 4.3. 

### 3.3. The Number of Graphene Layers and Defect Density

**Figure 7:**
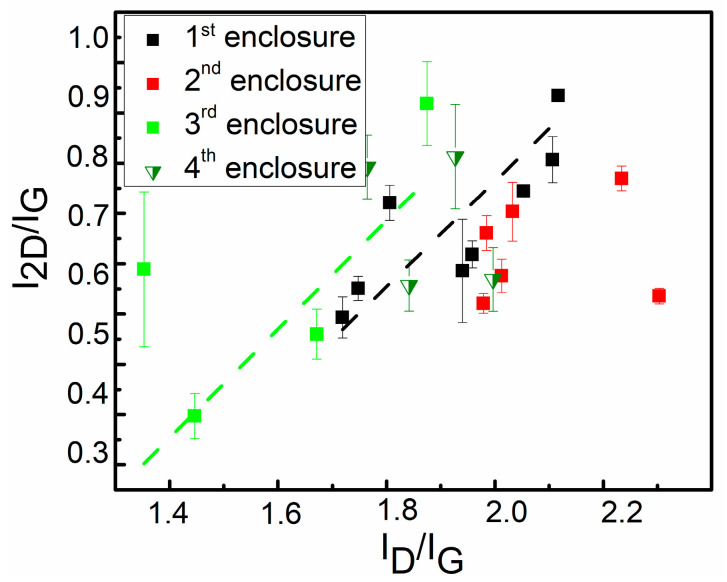
I_2D_/I_G_ vs. I_D_/I_G_ plot for all investigated samples. Dash lines represent the observed linear distributions.

Figure 7 shows the I_2D_/I_G_ vs. I_D_/I_G_ ratio plot for all investigated samples (Table 1). It is considered that the I_2D_/I_G_ vs. I_D_/I_G_ ratio change for the 1st and the 3rd enclosure followed linear distribution pattern as the I_2D_/I_G_ increased with I_D_/I_G_ ratio. Such an outcome contradicts the results reported by [24] (Table S1), where I_2D_/I_G_ decreased as a result of oxygen ion etching. It can be explained by the different nature of the defects in our study and [24] (boundary defects vs. irradiation defects). 

No clear dependence of the I_2D_/I_G_ ratio on the enclosure configuration was found (Figure 7). The lowest I_D_/I_G_ ratios were observed for graphene synthesized using the 3rd enclosure, while for graphene samples synthesized using the 1st enclosure, the range of I_D_/I_G_ ratio values was the broadest. Almost all I_D_/I_G_ ratio values of the graphene synthesized using the 2nd enclosure were within the range typical for graphene grown using the 1st enclosure. Additionally, comparing the I_D_/I_G_ ratios of the graphene samples synthesized using the 1st and the 2nd enclosures and the same other synthesis conditions, one can see that in some cases larger I_D_/I_G_ ratio values were found for the 1st enclosure, and in other cases for the 3rd enclosure (Figure 4, Figure 5 and Figure 6). 

The enclosure design was simplified even more, considering the results described above, though less graphene synthesis experiments were performed using the 3rd enclosure. No holes at the protective enclosure’s center resulted in a decrease in the I_D_/I_G_ ratio (see Figure 7). Therefore, holes were removed from the enclosure’s entire surface to suppress the direct plasma effects further. The modified enclosure shape was simply a rectangular steel sheet folded in two places (Figure 1d). Graphene was synthesized at the temperature of 700 °C using a protective sheath of such a simple structure. The I_2D_/I_G_ and I_D_/I_G_ ratios of these graphene samples were within the typical values for the 3rd envelope, although no additional optimization of the deposition conditions was performed. Further detailed research on graphene synthesized using simplified enclosures is in progress.

### 3.4. Dopant Density and Stress

The 2D peak position dependence on the G peak position can provide information about the graphene’s doping and stresses in graphene layers. This was shown in numerous studies investigating single-layer graphene synthesized by CVD on catalytic copper foil and transferred to the target substrate [28,29,30,31,32,33]. Some studies on multilayer transferred graphene have also been carried out [30]. The reported results from different authors follow similar dependencies. However, in directly synthesized graphene, the defect density is usually higher [20]. Therefore, the D peak is visible in the directly synthesized graphene’s Raman scattering spectrum [20,21,22,38]. This makes the situation more complicated. Hence, an additional analysis was carried out regarding the possible influence of defects on other Raman scattering spectrum parameters. It should be noted that the I_2D_/I_G_ ratio depends on the defect density, concentration of dopants, and the number of graphene layers. It was shown that the I_2D_/I_G_ ratio decreases with the appearance of the defect-related D peak in the Raman spectrum and the subsequent increase in defect density [41]. Graphene doping also leads to a reduced I_2D_/I_G_ ratio [41]. However, in our case, opposite dependence was found (Figure 7).

Another structural parameter that can influence the analysis of graphene doping and stress level is the different number of graphene layers. Both the 2D and G peak positions depend on the number of graphene layers (Table S1). In some studies, the upshift of the 2D peak position has been shown for few-layer graphene [30]. Following the latter research work, Raman spectra of the transferred graphene and the relationship between Pos(2D) vs. Pos(G) plots and stress as well as doping were analyzed [30]. 

Figure S1 shows a shift from Pos(2D) to the higher wavenumbers with increasing I_2D_/I_G_ ratio. However, Pos(2D) should shift to the lower wavenumbers with increasing I_2D_/I_G_ ratio due to the decrease in the number of graphene layers [39]. In our case, no clear dependence of the I_2D_/I_G_ ratio on the G peak position was found (Figure S2). Thus, the Pos(2D) upshift should not be related to the increased number of graphene layers. 

**Figure 8:**
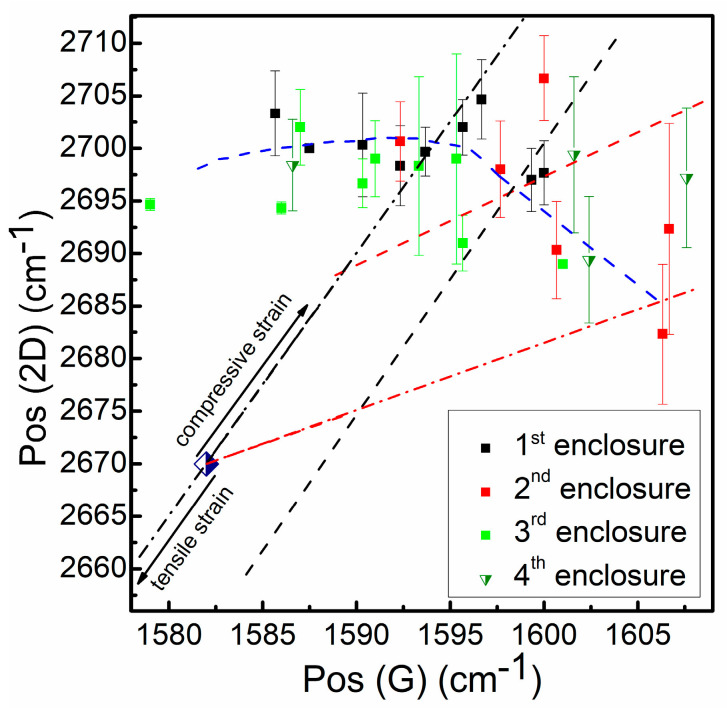
Pos(2D) vs. Pos(G) plot. The black dash-dot line refers to the undoped strained graphene (plotted according to the method [28]). The black dot line refers to the p-type doped strained graphene (constant hole concentration and different stress levels) (plotted according to [28]). The red dash-dot line refers to the unstrained p-type graphene (plotted according to [28]). The red dot line refers to the p-type doped strained graphene (constant stress level and different hole concentrations) (plotted according to the method [28]). The blue dash line refers to the strained n-type doped graphene (plotted according to [30], taking into account graphene layer number related shift of 2D peak position). The navy and white colored rhombus symbol refers to the unstrained and undoped graphene [28].

The dependence of Pos(2D) on Pos(G) is shown in Figure 8. Both the 2D and the G peak positions are shifted to the higher wavenumbers than the values typical for single-layer defect-free graphene. A vector analysis of the plot was performed according to the methodology presented in [28,29,30,31,32,33]. It can be seen that the 2D peak position is significantly shifted to the higher wavenumbers (by ~25–35 cm^−1^) compared to a value typical for defect-free, undoped, transferred single-layer graphene. The graphene samples synthesized in the present study were determined to be in the range of 2–4 layers thick, according to the analysis of the I_2D_/I_G_ ratio (see Figure 7 and Figure S3, for calculation method, see [23] and Table S1). However, the 2D peak position values are also shifted upwards compared to the position typical for two-layer defect-free undoped transferred graphene. Thus, it is considered that compressive stress was present in the investigated graphene samples (according to [28,29,30,31,32,33] and Table S1), in agreement with [42]. 

The analysis of the plot (Figure 8, Figure S4) regarding possible doping of the graphene revealed a less convenient picture. It should be emphasized that p-type graphene was investigated in most of the studies mentioned above [28,29,30,31,33]. The results reported in [28] revealed unintentional p-type doping of the graphene. However, the overall dependence of Pos(2D) on Pos(G) does not follow the vectors typical for p-type doped graphene (Figure 8, Figure S4). This behavior is rather typical for the n-type doped and strained graphene [30]. 

### 3.5. AFM Study

Several graphene samples synthesized using the 4th protective enclosure were studied by AFM to determine the number of the graphene layers as well as to evaluate the continuity of the graphene. AFM topographical images of the samples are presented in Figures S5–S7. The graphene samples’ surface morphology is very different from silicon substrate morphology (Figure S8). In all cases, AFM images revealed continuous horizontal graphene layers (Figures S5–S7). Similar AFM images were reported for directly synthesized graphene by other authors (e.g., [20,21,44,45]). One can notice some black features corresponding to the lowest surface points. It can be interpreted as holes in the graphene [46]. 

Previous studies [47,48,49] reported that step height of a single-layer graphene was found to be in the range of ~0.35–0.4 nm. The graphene AFM height profile was analyzed. Approximate graphene thickness was calculated by measuring the height from the zero points corresponding to the graphene holes to the profile maxima. A mixture of the single-layer and two-layer graphene was found for samples 1E4 and 2E4 (Figures S5–S7). In the case of the sample 3E4, up to three graphene layers can be found. An alternative graphene thickness evaluation method by using the AFM measurement data was performed following the histogram method [46,48]. Hence, the influence of the adsorbed contaminants can be minimized. According to the histogram method, the thickness of the graphene was between one and two layers for samples 1E4 and 3E4, while the thickness of sample 2E4 corresponded to single-layer graphene (Table S2, and Figures S5b, S6b, S7b and S8b). Thus, the average graphene thickness was found to be between one and two layers. In this case, graphene thickness evaluated via the use of AFM measurement data is in good agreement with graphene thickness calculated from the I_2D_/I_G_ ratio according to [23] (Figure S3).

## 4. Discussion

### 4.1. Effect of the Deposition Conditions. Comparison with Results Reported Elsewhere

Our obtained results were further compared with previous studies. In most of the studies regarding direct graphene synthesis, graphene was grown on different dielectric substrates. There are substantially less studies reporting direct graphene synthesis on silicon. Therefore, graphene’s direct growth on different substrates was considered in this comparison. 

It must be pointed out that in [38], no clear dependence of the I_2D_/I_G_ ratio on the process time was found for graphene directly synthesized on dielectric SiO_2_, quartz, and sapphire substrates from a CH_4_/H_2_/Ar gas mixture. Nevertheless, an I_D_/I_G_ ratio increase with the process time was observed, in good agreement with the present research. 

There are few studies on the effects of the hydrocarbon gas and hydrogen flow ratios on the structural properties of directly synthesized graphene. Analogously to our research, the I_2D_/I_G_ and I_D_/I_G_ ratios decreased with increasing C_2_H_2_/H_2_ flow ratio for graphene directly synthesized on fused silica and quartz by electron cyclotron resonance (ECR) PECVD [44]. A similar tendency was found in [45], where direct graphene synthesis on quartz via the CVD process was performed. Following the latter work, a too low methane flow resulted in no graphene growth, in agreement with our results. However, the I_D_/I_G_ ratio increased with the increase in CH_4_/H_2_ flow ratio [45]. 

Similarly to the graphene synthesized using the 1st enclosure, the I_2D_/I_G_ ratio increased with plasma power for graphene directly synthesized via PECVD on SiO_2_ [50]. However, the I_D_/I_G_ ratio in that study decreased with plasma power [50]. 

Likewise to the graphene synthesized in our study using the 1st and the 2nd enclosure, the I_2D_/I_G_ ratio increased with process temperature for graphene synthesized on Si(100) and glass from a C_2_H_2_/Ar gas mixture via microwave PECVD [51]. An opposite result was reported for graphene grown on quartz and fused silica substrates from a C_2_H_2_/H_2_ gas mixture via ECR PECVD [44]. In in [50] and [21], I_2D_/I_G_ ratio dependence on temperature was rather non-monotonic. The I_2D_/I_G_ ratio was highest [21] or lowest [50] at the specific temperature range used for the synthesis of graphene. Similarly to the graphene synthesized using the 1st enclosure, the I_D_/I_G_ ratio increased with temperature in [50]. A decrease in the I_D_/I_G_ ratio with temperature was reported in [21,44,51], similar to the case of the graphene synthesized using the 2nd enclosure. 

Thus, the different effects of the gas flow ratio, plasma power, and temperature on the graphene structure were found in various studies. The discrepancy of the results reported by other authors was more considerable than the results reported in the present study. 

### 4.2. Effect of the Deposition Conditions. Physical and Chemical Phenomena Involved

The graphene structure’s dependence on the technological synthesis process conditions found in the present research can be explained by considering the main graphene growth-related physical and chemical processes. 

Notably, the decrease in the number of graphene layers with increasing process time can be explained by the hydrogen etching prevailing over the growth of additional graphene layers as it was reported in [52]. 

The increase in the graphene I_2D_/I_G_ ratio with plasma power (Figure 4) can be related to the dependence of the methane and hydrogen dissociation rate on plasma power [50]. It can be considered that, in the case of graphene grown using the 1st enclosure, carbon-containing reactive species concentration increased with plasma power slower than hydrogen atoms and ion concentration, while in other cases, changes in that concentration with plasma power were non-monotonic. It seems that graphene defect density increased with plasma due to the decrease in the graphene crystallite size or the enhanced irradiation by ions and electrons. 

The decrease in the number of graphene layers with increased CH_4_/H_2_ gas flow ratio was observed in Figure 5. It can be explained by competition between two processes: graphene growth due to the carbon-containing active species flux towards the surface [50,53] and etching of the carbon-carbon bonds by hydrogen [44,54,55]. If the CH_4_/H_2_ ratio is too low, the etching reaction is much faster than the growth of the graphene layers [56]. This is the reason why no graphene growth was observed for samples 3E1, 4E2, and 4E3. The increase in the methane to hydrogen gas flow ratio resulted in a suppression of graphene growth over the etching. In this case, a further increase in the active flux of CH_x_ and C species towards the substrate and the reduced amount of hydrogen atoms will result in the increase in the number of graphene layers. The increasing size of graphene nanocrystals cannot explain the reduction in the I_D_/I_G_ ratio with the increasing CH_4_/H_2_ ratio presented in Figure 5. It is because the increase in graphene nucleus density with decreased H_2_ content due to the hydrogen etching resulted in a smaller graphene grain size [44]. Thus, the possible lowering of the hydrogen plasma-induced defect density with decreased hydrogen gas flow should be considered [57]. 

Several phenomena should be taken into account to explain the lowering of the number of graphene layers with increased process temperature (Figure 6). It was reported in [44] that the rate of the graphene etching by hydrogen decreases with temperature [44]. Therefore, it should instead result in a decrease in the I_2D_/I_G_ ratio with temperature. On the other hand, the desorption rate of carbon atoms increases with temperature, as reported in [52]. This can be a cause of the decrease in the number of graphene layers with increased deposition temperature. 

### 4.3. Effect of Protective Enclosure Design

A few studies could be considered while analyzing the protective enclosure design’s influence on the graphene structure. Notably, a study on graphene synthesized on glass using a copper foam-based protective Faraday cage revealed a tendency that a smaller aperture hole size can result in a better electric field shielding effect [22]. Thus, it can be considered that in our case, the lower I_D_/I_G_ ratios for graphene synthesized using the 3rd protective enclosure can be explained by better suppression of the electric field due to the absence of the holes at the enclosure’s center. On the other hand, no apparent difference between the I_D_/I_G_ ratios of the graphene synthesized using the 1st and the 2nd protective enclosures is in good accordance with [22], where reduced protective cage hole size resulted in no further apparent decrease in electric field strength.

### 4.4. Mechanisms Responsible to the N-Type Self Doping of Graphene and Induction of Compressive Stress

According to Figure 8 and Figure S4, unintentional n-type doping was found for directly synthesized graphene. It is worth noting that self-induced doping was already observed for graphene directly synthesized on Ge(111) by CVD [58]. Possible sources of such behavior would be adsorbates or substrate-induced effects. Atmospheric adsorbates (oxygen, water) usually result in p-type doping of the graphene [59,60,61]. P-type self-doping for graphene transferred onto the SiO_2_ substrate occurred due to various surface treatments and residual charges created on the substrate [62]. Unintentional graphene n-type doping was reported for epitaxial graphene directly synthesized on SiC via high-temperature annealing in a vacuum or inert ambient gas [63,64]. It was explained by the substrate-to-graphene charge transfer [64]. A study of the graphene transferred onto different substrates revealed that p-type self-doping, n-type self-doping, and no doping could be achieved via selection of the appropriate substrate [58,65]. Simulations revealed that when graphene is put onto SiO_2_, the graphene’s electronic structure strongly depends on the interface geometry and surface polarity [66]. In the case of the O-polar SiO2 surface with dangling bonds, graphene’s p-type doping takes place [66]. Graphene placing on the Si-polar surface with dangling bonds results in graphene’s n-type self-doping [66]. Considering the studies mentioned above, it is supposed in our case that charge transfer from the Si(100) substrate to the graphene took place during the microwave PECVD process. It resulted in the n-type self-doping of the graphene, similar to the cases of SiC and Si-polar SiO_2_ substrates. 

One can see in Figure 8 that directly synthesized graphene in the present study is found to be stressed. This stress is compressive in nature. It should be mentioned that, in the case of the exfoliated graphene transferred onto SiO_2_, pristine graphene sheets may exhibit both compressive and tensile strain [28]. This native strain becomes compressive due to the annealing at 100 °C or higher temperatures [28]. Similar effects of the annealing were reported for CVD synthesized graphene transferred onto the SiO_2_ substrate [67]. On the other hand, epitaxial graphene directly grown on SiC above 1100 °C exhibited substrate-induced compressive strain [28]. Compressive stress may be present in graphene directly synthesized on Si(100) [42], quartz [44], Ge(110) [58], and SiO_2_ [68]. Thus, our results are in good agreement with the studies mentioned above. It is considered that, in our case, compressive stress was induced during a direct graphene synthesis as thermal stress due to the large lattice mismatch between graphene and Si, as was suggested in [20,69].

## 5. Conclusions

In conclusion, the transfer-less and catalyst-less synthesis of graphene on Si(100) substrates via a combination of direct microwave plasma-enhanced chemical vapor deposition and protective enclosures was performed.

A study of the effect of the CH_4_/H_2_ gas flow ratio, temperature, and plasma power on graphene structure revealed that the most significant technological parameter used in the present study was methane and hydrogen gas flow ratio. Plasma power effects were the least pronounced. It seems that if a temperature is sufficiently high for graphene synthesis, the crucial process is a competition between plasma etching by hydrogen and carbon-containing active species influx towards the surface. If hydrogen flow is too high and/or methane flow is too low, etching prevails against growth and no graphene is formed. Afterwards, the number of graphene layers increases with carbon species flow and/or with decreased hydrogen flow. Hydrogen species density is a much more critical etching factor than increased plasma power. The thermally stimulated desorption of carbon atoms is important, while the formation of the plasma-induced radiation defects has less influence on graphene growth and defect density. 

A study of the enclosure effects revealed no top hole size effects for investigated enclosures. The absence of top holes in the middle of the enclosure reduced the plasma effect on the growing graphene and decreased defect density. The graphene was successfully synthesized using just a rectangular steel sheet folded in two places as a simplified protective enclosure, considering these results. Graphene was grown at a temperature of 700 °C using a protective sheath with such a simple structure. 

Analysis of the positions of 2D and G peaks revealed unintentional n-type doping of the graphene. It was explained by charge transfer from the Si(100) substrate to the graphene. The presence of compressive stress was found in graphene. It was supposed that the large lattice mismatch between the growing graphene and the silicon induced thermal stress.

An atomic force microscopy study confirmed the growth of continuous horizontal graphene layers. 

## Figures and Tables

**Table 1:** Graphene synthesis conditions used in the present study.

Sample No.	Enclosure No.	P, kW	H_2_, sccm	CH_4_, sccm	p, mBar	t, °C	t, min
2E1	1	1.2	150	50	30	900	30
3E1	1	1.2	180	20	30	900	30
4E1	1	1.2	120	80	30	900	30
5E1	1	1.2	150	50	30	900	15
6E1	1	1.2	150	50	30	900	45
7E1	1	1.2	150	50	30	700	30
8E1	1	1.2	150	50	30	800	30
9E1	1	0.8	150	50	30	900	30
10E1	1	1.0	150	50	30	900	30
1E2	2	1.2	150	50	30	800	30
2E2	2	1.2	150	50	30	700	30
3E2	2	1.2	150	50	30	900	30
4E2	2	1.2	180	20	30	900	30
5E2	2	1.2	120	80	30	900	30
6E2	2	1.0	150	50	30	900	30
7E2	2	0.8	150	50	30	900	30
3E3	3	1.2	150	50	30	900	30
4E3	3	1.2	180	20	30	900	30
5E3	3	1.2	120	80	30	900	30
6E3	3	0.8	150	50	30	900	30
7E3	3	1.0	150	50	30	900	30
1E4	4	1.2	150	50	30	800	30
2E4	4	1.2	150	50	22	700	30
3E4	4	1.2	150	50	22	800	30
4E4	4	1.2	150	50	22	900	30

## Supplementary Materials

The following are available online at https://www.mdpi.com/1996-1944/13/24/5630/s1, Table S1: Possible relations of the Raman scatterings spectra parameters mentioned above the number of graphene layers, stress, doping and defect density, Table S2: Graphene samples and silicon substrate surface roughness histogram peak maximums and graphene thickness values according to the histogram method, Figure S1: Pos(2D) Vs I2D/IG plot, Figure S2: I2D/IG Vs Pos(G) plot, Figure S3: I2D/IG ratio of samples 4E1, 4E2, 4E3 and number of the graphene layers calculated according to [23], Figure S4: Pos(2D) vs. Pos(G) plot for sample 1E4, Figure S5: AFM image (**a**), height distribution histogram (**b**) and height profile (**c**) of the graphene sample No 1E4, Figure S6: AFM image (**a**), height distribution histogram (**b**) and height profile (**c**) of the graphene sample No 2E4, Figure S7: AFM image (**a**), height distribution histogram (**b**) and height profile (**c**) of the graphene sample No 3E4.
