# Supplementary material for: Catalyst-Less and Transfer-Less Synthesis of Graphene on Si(100) Using Direct Microwave Plasma Enhanced Chemical Vapor Deposition and Protective Enclosures
Source: Materials (Basel). 2020 Dec 10;13(24):5630. doi: 10.3390/ma13245630 (PMC7763619; doi:10.3390/ma13245630)
Supplement: Supplementary file 1 [file materials-13-05630-s001.pdf]

materials

Supplementary Materials

Catalyst-Less and Transfer-Less Synthesis of Graphene on Si(100) Using Direct Microwave Plasma Enhanced Chemical Vapor Deposition and Protective Enclosures

Rimantas Gudaitis, Algirdas Lazauskas, Šarūnas Jankauskas and Šarūnas Meškinis *

Table S1. Possible relations of the Raman scatterings spectra parameters mentioned above with the number of graphene layers, stress, doping, and defect density. For references please see references list of the manuscript.

Parameter of the Graphene Raman Scattering Spectra	Number of Graphene Layers (n)	Stress	Doping (p-Type)	Doping (n-Type)	Defects
I_{2D}/I_G	Decrease with layer number by a law 0.63-0.0595·n (for n>1) [1] Decrease with layer number [2]		Decrease with doping [3].	Decrease with doping [4].	Decrease with defects density [5]
I_D/I_G	N.d.				Increase up to 4 and then decrease [5]
Position of G peak	1587−1.34·n (for n>1) [1]	Shift to the higher wavenumbers with compressive stress [6–11] Shift to the lower wavenumbers with tensile stress [12–16]	Shifts to the higher wavenumbers with increased dopant density [7–10,12]	At the first no clear shift, afterward, shifts to the lower wavenumbers with increased dopant density [6,12]	
Position of 2D peak	2686.6+2.63·n (for n>1) [1]	Shift to the higher wavenumbers with compressive stress [6–11] Shift to the lower wavenumbers with tensile stress [12–15]	Shifts to the higher wavenumbers with increased hole density [7–12]	Shifts to the lower wavenumbers with increased electron density[6,12],	

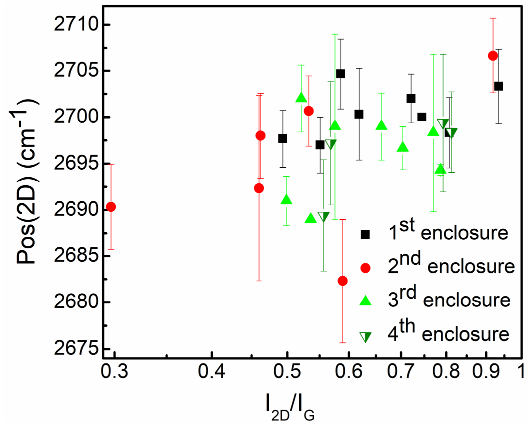

Figure S1. Pos(2D) Vs I_{2D}/I_G plot.

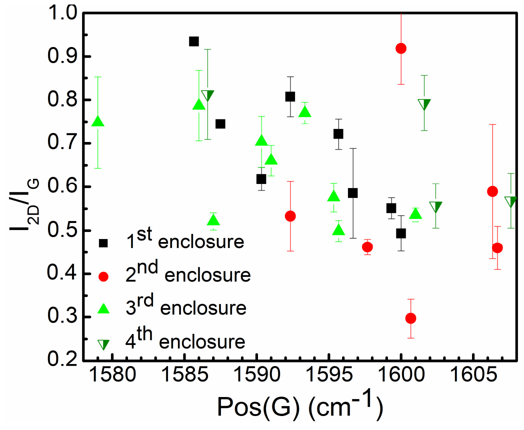

Figure S2. I_{2D}/I_G Vs Pos(G) plot.

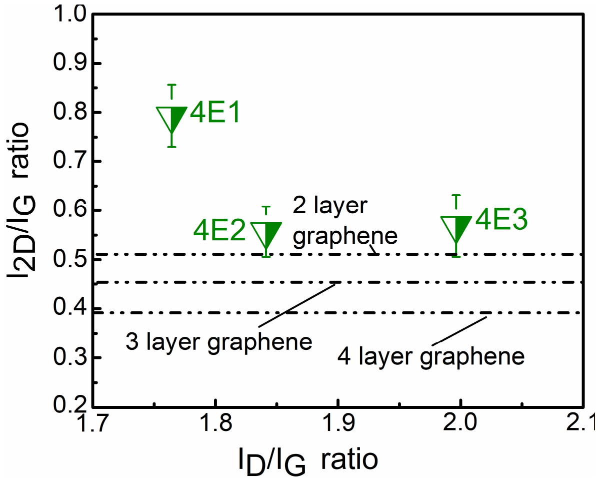

Figure S3. I_{2D}/I_G ratio of samples 4E1, 4E2, 4E3 and number of the graphene layers calculated according to [1].

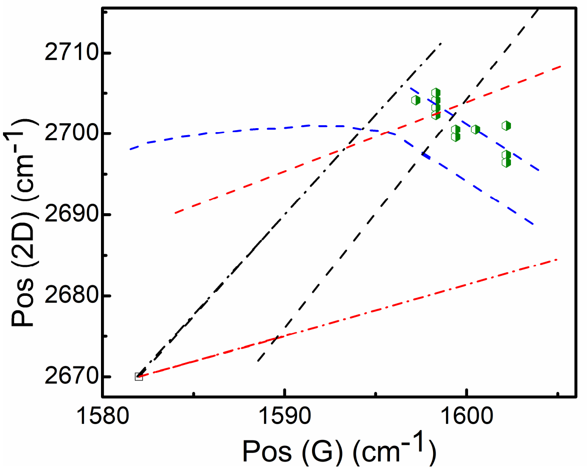

Figure S4. Pos(2D) vs. Pos(G) plot for sample 1E4. The black dash-dot line refers to the undoped strained graphene (plotted according to the method [6]). The black dot line refers to the p-type doped strained graphene (constant hole concentration and different stress levels) (plotted according to [6]). The red dash-dot line refers to the unstrained p-type graphene (plotted according to [6]). The red dot line refers to the p-type doped strained graphene (constant stress level and different hole concentrations) (plotted according to the method [6]). The blue dash line refers to the strained n-type doped graphene (plotted according to [8], taking into account graphene layer number related shift of 2D peak position). The hollow square symbol refers to the unstrained and undoped graphene [6].

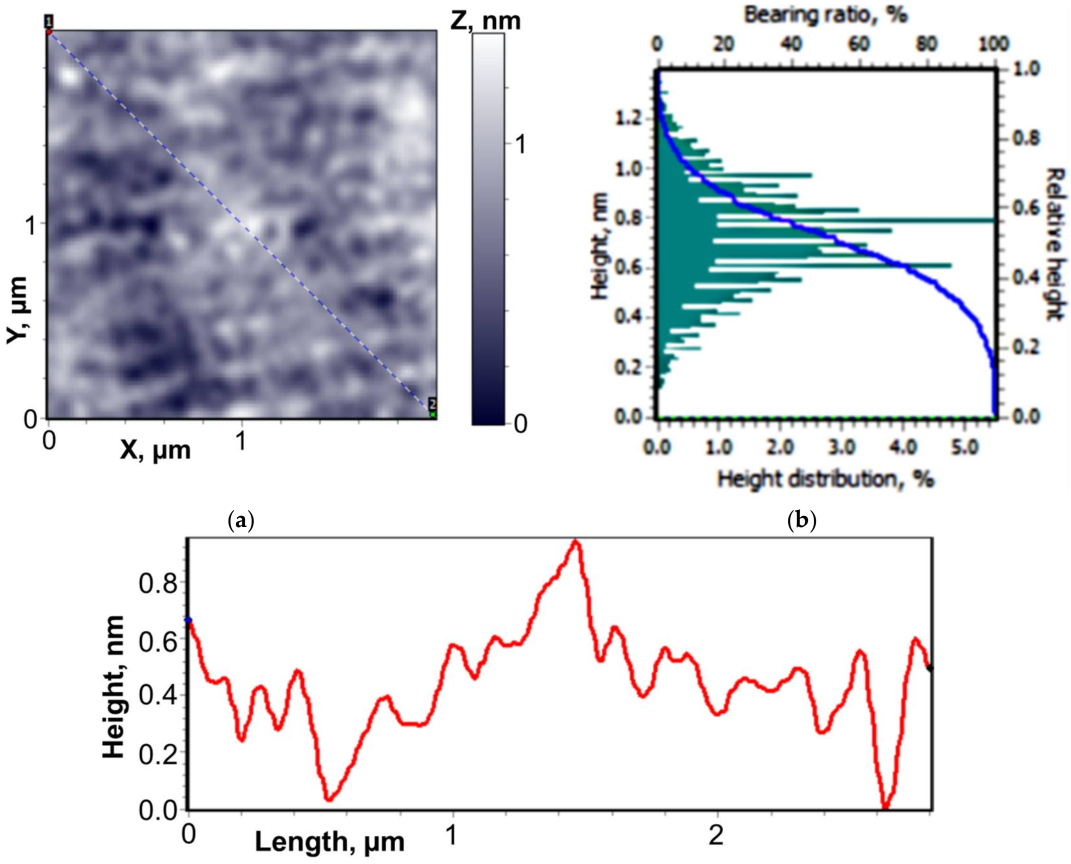

(c)

Figure S5. AFM image (**a**), height distribution histogram (**b**), and height profile (**c**) of the graphene sample No 1E4.

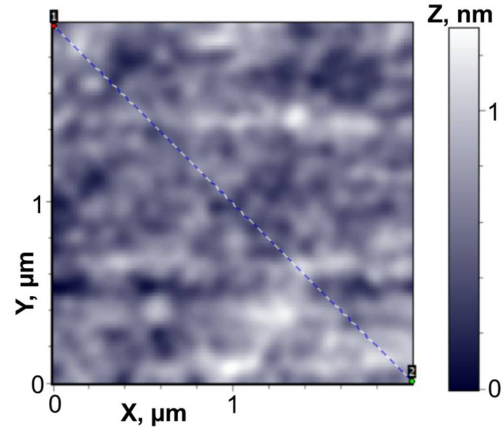

(a)

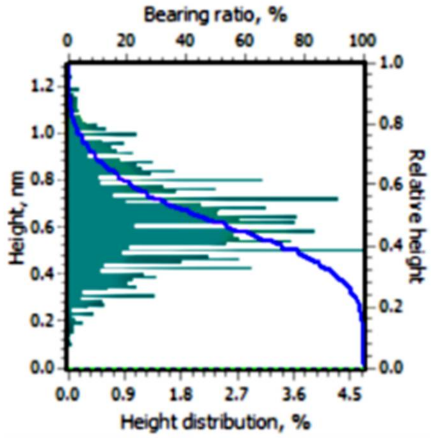

(b)

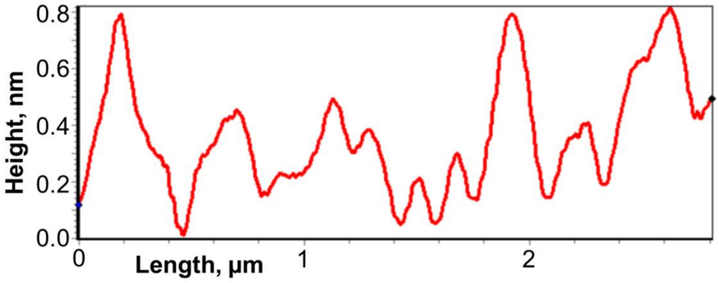

(c)

Figure S6. AFM image (**a**), height distribution histogram (**b**), and height profile (**c**) of the graphene sample No 2E4.

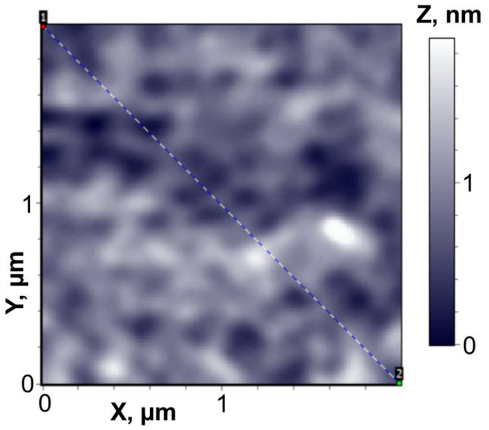

(a)

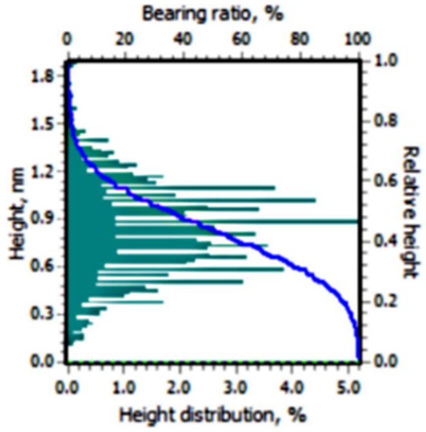

(b)

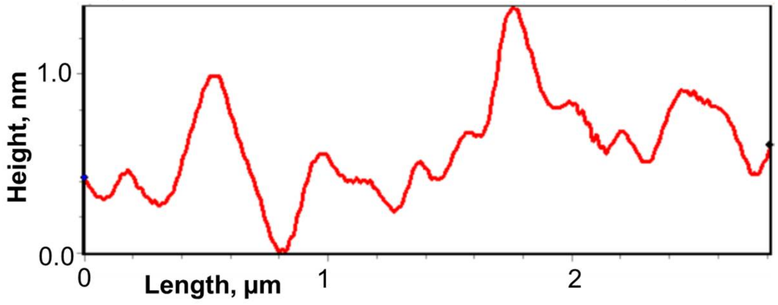

(c)

Figure S7. AFM image (**a**), height distribution histogram (**b**) and height profile (**c**) of the graphene sample No 3E4.

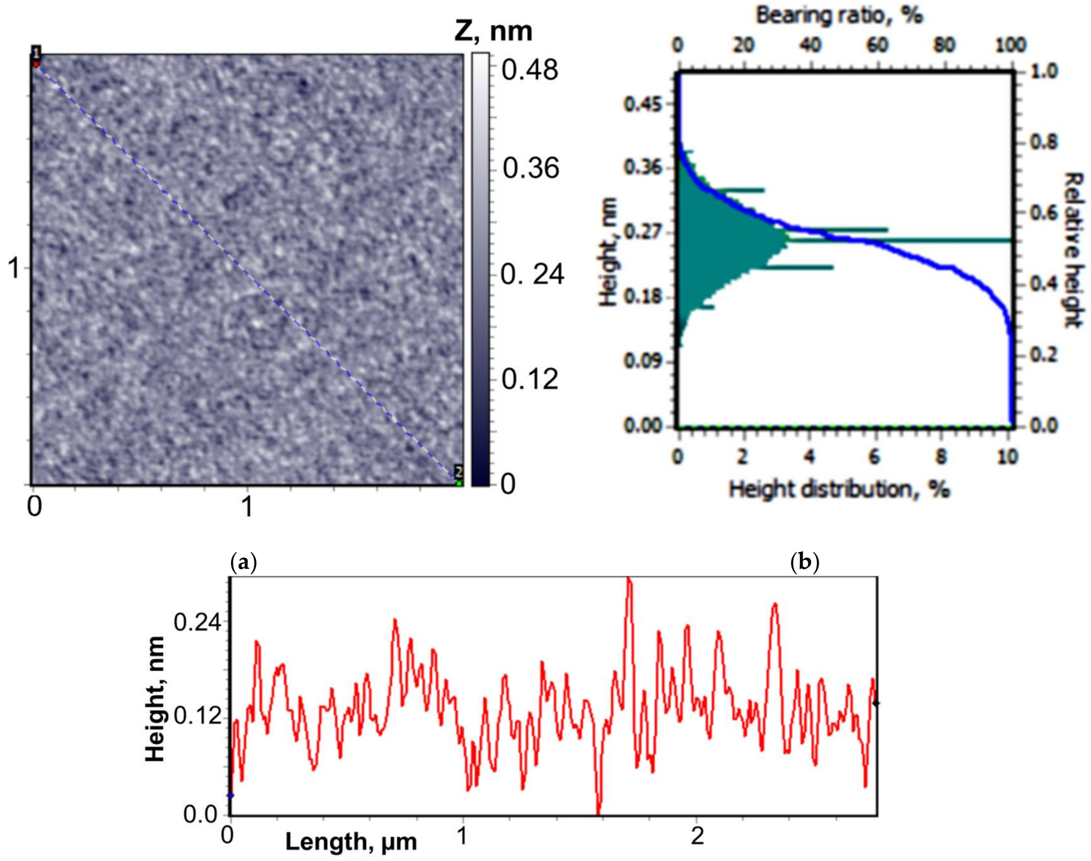

(**a**) (**b**)

(**c**)

Figure S8. AFM image (**a**), height distribution histogram (**b**), and height profile (**c**) of the monocrystalline silicon substrate.

Table S2. Graphene samples and silicon substrate surface roughness histogram peak maximums and graphene thickness values according to the histogram method [17,18].

Sample	Surface Roughness Histogram Peak Maximum (nm)	Graphene Thickness (nm)
1E4	0.74	0.47
2E4	0.62	0.35
3E4	0.83	0.56
Si(100) substrate	0.27	-

References

1. Hwang, J.-S.; Lin, Y.-H.; Hwang, J.-Y.; Chang, R.; Chattopadhyay, S.; Chen, C.-J.; Chen, P.; Chiang, H.-P.; Tsai, T.-R.; Chen, L.-C. Imaging layer number and stacking order through formulating Raman fingerprints obtained from hexagonal single crystals of few layer graphene. *Nanotechnology* **2012**, *24*, 015702, doi:10.1088/0957-4484/24/1/015702.
2. Ni, Z.H.; Wang, Y.Y.; Yu, T.; Shen, Z.X. Raman Spectroscopy and Imaging of Graphene. *Nano Res.* **2008**, *1*, 273–291, doi:10.1007/s12274-008-8036-1.
3. Zhao, W.; Tan, P.H.; Liu, J.; Ferrari, A.C. Intercalation of Few-Layer Graphite Flakes with $FeCl_3$: Raman Determination of Fermi Level, Layer by Layer Decoupling, and Stability. *J. Am. Chem. Soc.* **2011**, *133*, 5941–5946, doi:10.1021/JA110939A.
4. Szirmai, P.; Márkus, B.G.; Chacón-Torres, J.C.; Eckerlein, P.; Edelthalhammer, K.; Englert, J.M.; Mundloch, U.; Hirsch, A.; Hauke, F.; Náfrádi, B. et.al. Characterizing the maximum number of layers in chemically exfoliated graphene. *Sci. Rep.* **2019**, *9*, 19480, doi:10.1038/S41598-019-55784-6.

5. Childres, I.; Jauregui, L. A.; Tian, J.; Chen, Y. P. Effect of oxygen plasma etching on graphene studied using Raman spectroscopy and electronic transport measurements. *New J. Phys.* **2011**, *13*, 025008, doi:10.1088/1367-2630/13/2/025008.
6. Lee, J. E.; Ahn, G.; Shim, J.; Lee, Y. S.; Ryu, S. Optical separation of mechanical strain from charge doping in graphene. *Nat. Commun.* **2012**, *3*, 1024, doi:10.1038/NCOMMS2022.
7. Sakavičius, A.; Astromskas, G.; Bukauskas, V.; Kamarauskas, M.; Lukša, A.; Nargelienė, V.; Niaura, G.; Ignatjev, I.; Treideris, M.; Šetkus, A. Long distance distortions in the graphene near the edge of planar metal contacts. *Thin Solid Films.* **2020**, *698*, 137850, doi:10.1016/J.TSF.2020.137850.
8. Kim, S.; Ryu, S. Thickness-dependent native strain in graphene membranes visualized by Raman spectroscopy. *Carbon.* **2016**, *100*, 283–290, doi:10.1016/J.CARBON.2016.01.001.
9. Armano, A.; Buscarino, G.; Cannas, M.; Gelardi, F. M.; Giannazzo, F.; Schilirò, E.; Agnello, S. Monolayer graphene doping and strain dynamics induced by thermal treatments in controlled atmosphere. *Carbon.* **2018**, *127*, 270–279, doi:10.1016/J.CARBON.2017.11.008.
10. Neumann, C.; Reichardt, S.; Venezuela, P.; Drögeler, M.; Banszerus, L.; Schmitz, M.; Watanabe, K.; Taniguchi, T.; Mauri, F.; Beschoten, B. et al. Raman spectroscopy as probe of nanometre-scale strain variations in graphene. *Nat. Commun.* **2015**, *6*, 8429, doi:10.1038/NCOMMS9429.
11. Lee, U.; Han, Y.; Lee, S.; Kim, J.S.; Lee, Y.H.; Kim, U.J.; Son, H. Time Evolution Studies on Strain and Doping of Graphene Grown on a Copper Substrate Using Raman Spectroscopy. *ACS Nano.* **2020**, *14*, 919–926, doi:10.1021/ACSNANO.9B08205.
12. Wu, J.-B.; Lin, M.-L.; Cong, X.; Liu, H.-N.; Tan, P.-H. Raman spectroscopy of graphene-based materials and its applications in related devices. *Chem. Soc. Rev.* **2018**, *47*, 1822–1873, doi:10.1039/C6CS00915H.
13. Zeng, Y.; Lo, C.-L.; Zhang, S.; Chen, Z.; Marconnet, A. Dynamically tunable thermal transport in polycrystalline graphene by strain engineering. *Carbon* **2020**, *158*, 63–68, doi:10.1016/J.CARBON.2019.11.060.
14. Mohiuddin, T.M.G.; Lombardo, A.; Nair, R.R.; Bonetti, A.; Savini, G.; Jalil, R.; Bonini, N.; Basko, D.M.; Galiotis, C.; Marzari, N. et al. Uniaxial strain in graphene by Raman spectroscopy: G peak splitting, Grüneisen parameters, and sample orientation. *Phys. Rev. B.* **2009**, *79*, 205433, doi:10.1103/PHYSREVB.79.205433.
15. Ni, Z.H.; Yu, T.; Lu, Y.H.; Wang, Y.Y.; Feng, Y.P.; Shen, Z.X. Uniaxial Strain on Graphene: Raman Spectroscopy Study and Band-Gap Opening. *ACS Nano.* **2008**, *2*, 2301–2305.
16. Chugh, S.; Mehta, R.; Lu, N.; Dios, F.D.; Kim, M.J.; Chen, Z.H. Comparison of graphene growth on arbitrary non-catalytic substrates using low-temperature. *Carbon* **2015**, *93*, 393–399, doi:10.1016/j.carbon.2015.05.035.
17. Yao, Y.X.; Ren, X.X.; Gao, S.T.; Li, S. Histogram method for reliable thickness measurements of graphene films using atomic force microscopy (AFM). *J. Mater. Sci. Technol.* **2017**, *33*, 815–820, doi:10.1016/j.jmst.2016.07.020.
18. Zhou, L.Z.; Fox, L.; Włodek, M.; Islas, L.; Slastanova, A.; Robles, E.; Bikondo, O.; Harniman, R.; Fox, N.; Cattelan, M.; Briscoe, W.H. Surface structure of few layer graphene. *Carbon* **2018**, *136*, 255–261, doi:10.1016/j.carbon.2018.04.089.
